# Safety and precision of frontal trajectory of lateral habenula deep brain stimulation surgery in treatment-resistant depression

**DOI:** 10.3389/fneur.2023.1113545

**Published:** 2023-03-16

**Authors:** Zhiqiang Cui, Chao Jiang, Chunhua Hu, Ye Tian, Zhipei Ling, Jian Wang, Tengteng Fan, Hongwei Hao, Zhiyan Wang, Luming Li

**Affiliations:** ^1^Department of Neurosurgery, Chinese People's Liberation Army of China General Hospital, Beijing, China; ^2^College of Life and Health Sciences, Institute of Neuroscience, Northeastern University, Shenyang, Liaoning, China; ^3^National Engineering Research Center of Neuromodulation, School of Aerospace Engineering, Tsinghua University, Beijing, China; ^4^Chinese People's Liberation Army General Hospital Hainan Hospital Neurosurgery, Sanya, Hainan, China; ^5^Peking University Sixth Hospital, Peking University Institute of Mental Health, National Health Commission Key Laboratory of Mental Health (Peking University), National Clinical Research Center for Mental Disorders (Peking University Six Hospital), Beijing, China; ^6^Chinese Academy of Sciences Key Laboratory of Mental Health, Institute of Psychology, Chinese Academy of Sciences, Beijing, China; ^7^Precision Medicine and Healthcare Research Center, Tsinghua-Berkeley Shenzhen Institute, Tsinghua University, Shenzhen, China; ^8^International Data Group/McGovern Institute for Brain Research at Tsinghua University, Beijing, China; ^9^Institute of Epilepsy, Beijing Institute for Brain Disorders, Beijing, China

**Keywords:** lateral habenula, deep brain stimulation, frontal trajectory, safety, treatment-resistant depression

## Abstract

**Introduction:**

The lateral habenula (LHb) is a promising deep brain stimulation (DBS) target for treatment-resistant depression (TRD). However, the optimal surgical trajectory and its safety of LHb DBS are lacking.

**Methods:**

We reported surgical trajectories for the LHb in six TRD patients treated with DBS at the General Hospital of the Chinese People's Liberation Army between April 2021 and May 2022. Pre-operative fusions of magnetic resonance imaging (MRI) and computed tomography (CT) were conducted to design the implantation trajectory of DBS electrodes. Fusions of MRI and CT were conducted to assess the safety or precision of LHb DBS surgery or implantable electrodes locations.

**Results:**

Results showed that the optimal entry point was the posterior middle frontal gyrus. The target coordinates (electrode tips) were 3.25 ± 0.82 mm and 3.25 ± 0.82 mm laterally, 12.75 ± 0.42 mm and 13.00 ± 0.71 mm posterior to the midpoint of the anterior commissure–posterior commissure (AC-PC) line, and 1.83 ± 0.68 mm and 1.17 ± 0.75 mm inferior to the AC-PC line in the left and right LHb, respectively. The “Ring” angles (relative to the AC-PC level on the sagittal section plane) of the trajectories to the left and right LHb were 51.87° ± 6.67° and 52.00° ± 7.18°, respectively. The “Arc” angles (relative to the midline of the sagittal plane) were 33.82° ± 3.39° and 33.55° ± 3.72°, respectively. Moreover, there was small deviation of actual from planned target coordinates. No patient had surgery-, disease- or device-related adverse events during the perioperative period.

**Conclusion:**

Our results suggested that LHb-DBS surgery *via* frontal trajectory is safe, accurate, and feasible. This is an applicable work to report in detail the target coordinates and surgical path of human LHb-DBS. It has of great clinical reference value to treat more cases of LHb-DBS for TRD.

## 1. Introduction

Depressive disorders have a lifetime prevalence of 6.9% and have been estimated to be the second leading cause of years lived with disability in China (1, 2). Approximately 30% of patients with depression fail to respond to two or more standard antidepressants taken at adequate doses for adequate durations, which is recognized as treatment-resistant depression (TRD) (3). Deep brain stimulation (DBS) is a promising therapy for TRD. Clinical studies have assessed putative therapeutic effects of DBS in participants with TRD across several major brain targets, such as the subgenual cingulate gyrus, ventral anterior limb of the internal capsule, superolateral medial forebrain bundle, and lateral habenula (LHb) (4–7). However, several randomized controlled clinical trials have not shown the efficacy of DBS for TRD (8–10).

Studies on dysfunctional neuronal networks have demonstrated that the LHb plays an important pathophysiological role in depression. Clemm von Hohenberg et al. reported a causal link between LHb downregulation and reduction in DMN connectivity in an animal model of TRD (11). Hu et al. found that the number of bursting neurons increased in LHb in congenitally learned helpless rats (12). Moreover, the first case of LHb stimulation in a patient with TRD who underwent surgery for DBS reached complete remission (7). Recently, with the advancement of DBS equipment, changes in the electrophysiological characteristics of the LHb have been reported for several cases of LHb DBS for TRD (13–15). These results suggest that the LHb is a potential target of DBS in the treatment of TRD.

The habenula (Hb) is a phylogenetically old structure located in the dorsomedial portion of the thalamus and comprises LHb and medial habenula portions (16, 17). The entire Hb has a volume of only ~30 mm^3^ and is located below the third ventricle. With the progress of imaging, the Hb has be identified in humans through high-resolution structural MRI (17). Although Schneider et al. reported that the enlarged ventricles and the superior thalamic vein were major trajectory obstacles of LHb (18, 19), there are occasional adverse events during actual DBS surgery, especially intracerebral hemorrhage. Considering the position and its small size of Hb, there pose challenges in ensuring the target location and optimal surgical trajectory and its safety in LHb DBS. Here, we reported the surgical trajectories of LHb DBS in six TRD patients, including the target coordinates, the trajectory design, and the accuracy and safety of LHb DBS surgery, providing clinical evidence for the surgical path using LHb as the target of DBS.

## 2. Methods

### 2.1. Patients

Six patients with TRD (one bipolar and five unipolar TRD patients) who underwent bilateral LHb DBS surgery from April 2021 to May 2022 at the First Medical Center, General Hospital of the Chinese People's Liberation Army were analyzed (Chinese Clinical Trials 2100045363). All patients gave the informed consents for participation in the clinical trial, which was approved by Chinese People's Liberation Army General Hospital Medical Ethics Committee. The enrolled patients met the following inclusion criteria: (1) 18–70 years of age, male or female; (2) meet the Diagnostic and Statistical Manual of Mental Disorders, 5th Ed (DSM-5th) criteria for major depressive disorder (MDD); (3) lack of antidepressant response to a minimum of two adequate antidepressant medications from different classes and other therapies, including psychotherapy, or electroconvulsive therapy and so on; (4) the 17-item Hamilton Depression Rating Scale (HDRS-17) total score ≥ 20; (5) The patient himself and his/her legal guardian can fully understand the therapy and agree to sign the informed consent form. The characteristics of these TRD patients treated with DBS were described in Table 1.

**Table 1:** Characteristics of 6 TRD patients treated with bilateral LHb DBS.

**Case**	**Age (y)**	**Male/Female**	**Diagnosis**	**Disease duration**	**Other psychiatric comorbidities**	**Anti-depressant medication**	**Preoperative HDRS-17 scores**
1	30	F	Unipolar TRD	22	/	SSRI, SNRI, CAM	35
2	22	M	Unipolar TRD	6	/	SSRI, SNRI, CAM	27
3	34	F	Unipolar TRD	2	/	SSRI, SNRI, CAM	32
4	18	F	Bipolar TRD	5	/	SSRI, SNRI, CAM	25
5	23	F	Unipolar TRD	7	/	SSRI, SNRI, CAM	25
6	37	M	Unipolar TRD	9	/	SSRI, SNRI, CAM	27

SSRI, selective serotonin reuptake inhibitors; SNRI, Serotonin and norepinephrine reuptake inhibitors; CAM, Complementary or alternative drug therapy; HDRS-17, 17-item Hamilton Depression Rating Scale; TRD, treatment resistant depression.

### 2.2. Parameters of MRI scan

Images of preoperative magnetic resonance imaging (MRI) were obtained with a 3.0T MRI device (MAGNETOM Espree, Siemens Healthineers, Erlangen, Germany). The MRI sequences and parameters included a T1-weighted three-dimensional (3D) magnetization-prepared rapid gradient echo (MP-RAGE) with a time to echo (TE) of 3.02 ms, repetition time (TR) of 1,650 ms, matrix size of 256 × 256, field of view (FOV) of 260 × 260 mm, FOV phase of 100%, slice thickness of 1 mm, and 16-cm slab and T2-weighted imaging with a TE of 93 ms, TR of 5,500 ms, matrix size of 512 × 512, FOV of 260 × 260 mm, FOV phase of 100%, and slice thickness of 3 mm. The diffusion tensor imaging (DTI) was with a TE of 79 ms, TR of 8,900 ms, FOV of 200 × 200 mm, FOV phase of 100%, radiant directions = 30, *b* = 3,000 s/mm^2^, and slice thickness of 2 mm.

### 2.3. Design principles of target and path

**Figure 1:**
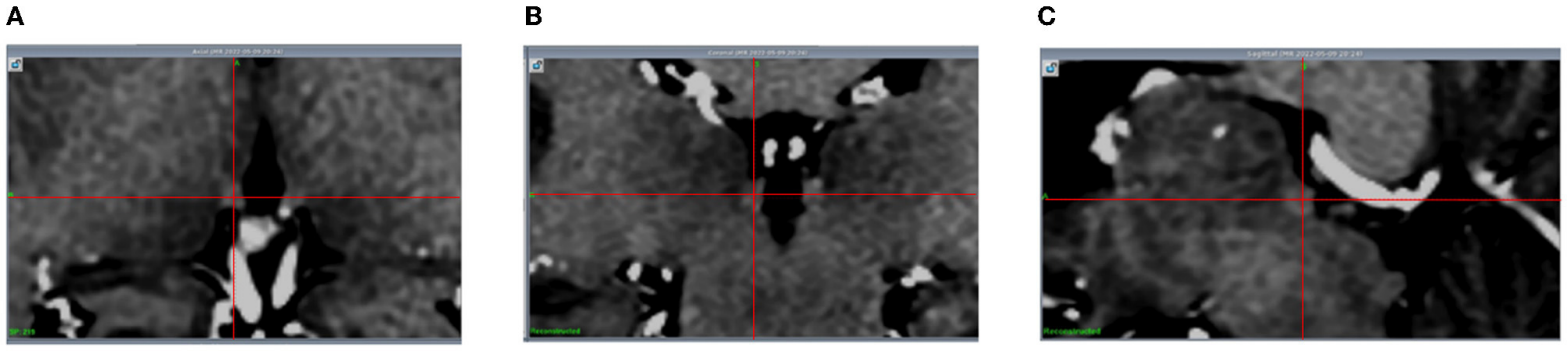
The Hb is visible (with the intersection of the red lines indicating the center of the left Hb) in 3D T1-weighted MR images. **(A)** Axial; **(B)** Coronal; **(C)** Sagittal.

**Figure 2:**
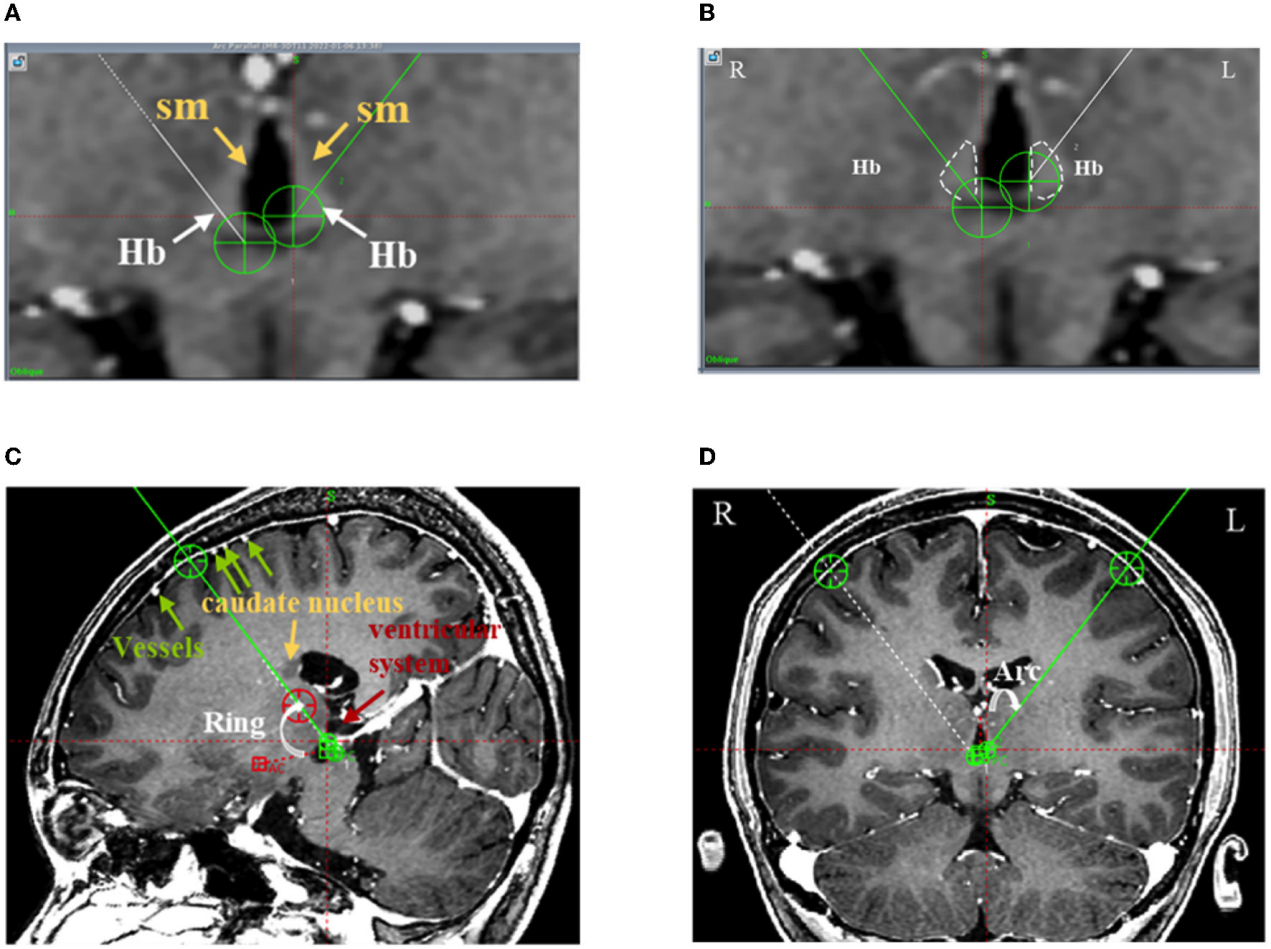
The frontal trajectory to the left LHb was planned using 3D T1 angiography images in Case 4. **(A)** The Hb and its afferent fiber tract (stria medullaris, sm) clearly distinguished from the surrounding gray matter and cerebrospinal fluid in the T1-weighted image; **(B)** The bilateral Hb delineated in white and the DBS electrode targeting the center of the left Hb; **(C)** The entry point of the planned frontal trajectory on the coronal plane, avoiding the cortical vessels, caudate nucleus, and ventricular system. Additionally, the planned frontal trajectory to the left side was at an angle of 59.10° relative to the AC-PC level on the sagittal plane (Ring); **(D)** The planned frontal trajectory to the left side was at an angle of 37.70° relative to the midline of the sagittal plane (Arc).

Regarding the target coordinates, the Hb locates above dorsal thalamus, which is a small nucleus. The Hb and its afferent fiber tract (stria medullaris, SM) are visible in 3D T1-weighted magnetic resonance (MR) images (Figures 1, 2A). Despite their small size, the 3D T1-weighted images can be used to plan the Hb target coordinates. Considering the frontal trajectory, as many contacts of the electrode as possible are in contact with the nucleus, and the target coordinates (electrode tips) are located at the posterior medial side of the Hb (Figures 2A, B).

Regarding the path, first, because the Hb and its fibers form a narrow, slender structure, the path was planned such that the electrode contacts pass through the long axis of the Hb as much as possible (Figure 2B); second, the trajectory was planned to avoid the anterior caudate, ventricular system, and pyramidal tract, guaranteeing the safety of the implantable trajectory (Figures 2C, D); and third, the entry point was planned so as to avoid the cortical vessels and the end point was planned so as to avoid the superior thalamic vein (Figures 2C, D).

### 2.4. DBS surgery

On the day of surgery, a model G stereotactic head frame (Leksell; Elekta, Stockholm, Sweden) was positioned on the patient. Computed tomography (CT) was performed and the results fused with those of the pre-surgical MRI. According to the locations of the left and right LHb in each patient, a safe frontal trajectory was planned for the left LHb and right LHb of each patient.

**Figure 3:**
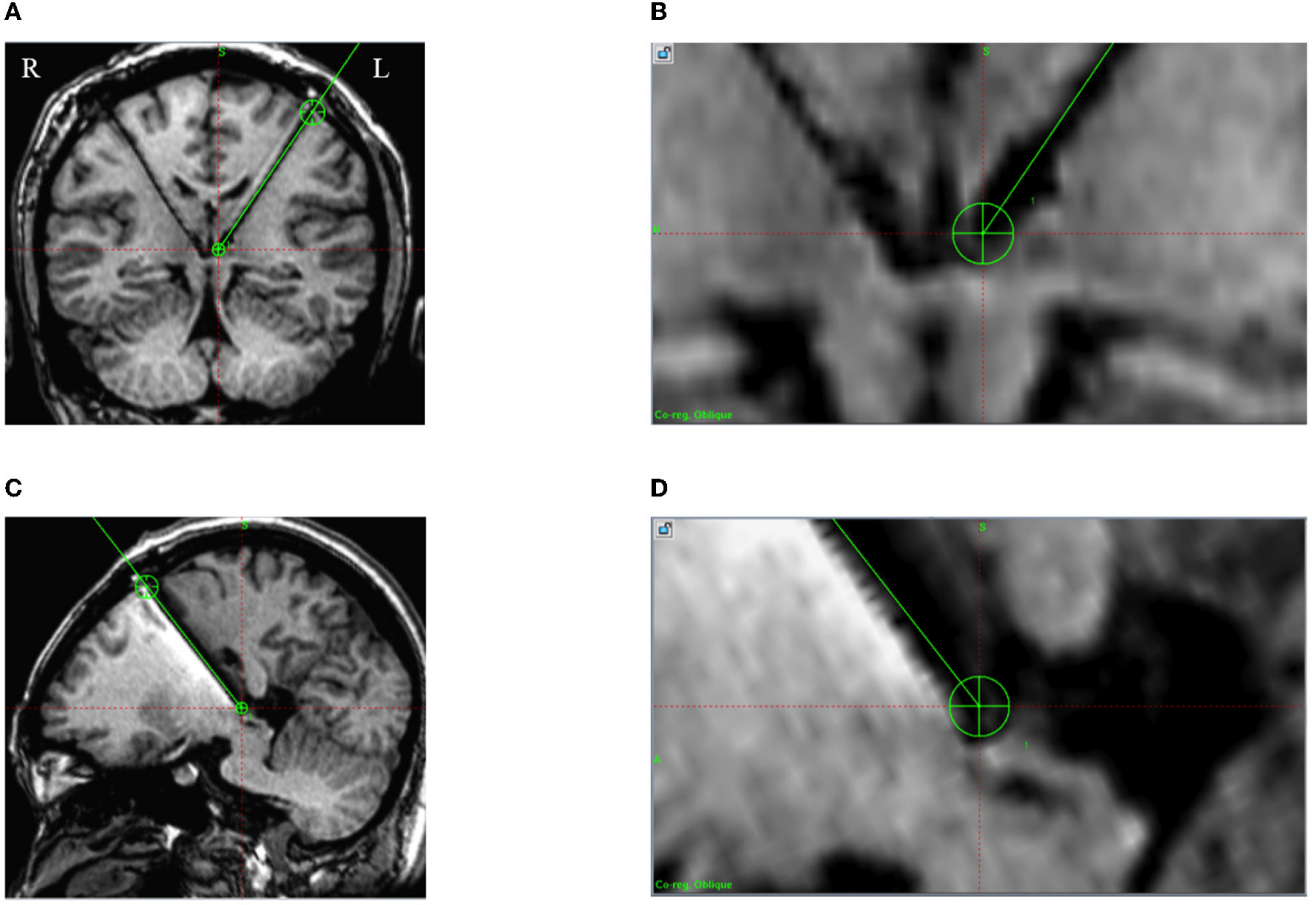
Intraoperative 3D T1 images in case 5. A green line is the preoperative planning trajectory, and the dark signal shows the positions of the electrode and lead. **(A, B)** The actual implanted DBS electrode consistent with the preoperative plan on the coronal plane; **(C, D)** The actual implanted DBS electrode consistent with the preoperative plan on the sagittal plane.

All surgery procedures were carried out with general anesthesia and the patient in the supine position. According to the predefined frame coordinates, the entry points of the scalp incision and skull drilling were determined, avoiding the cerebral cortex sulci and ventricle and cortical vessels. Two holes were then drilled continuously either side of the head. The coordinates of the orientation instrument were adjusted to guide pin points to the bone holes. Immediately after the dura was incised and the trocar was inserted, fibrin sealing gel was injected into the bone hole. In reducing the leakage of cerebrospinal fluid, the neck was flexed to raise the head as far as possible while maintaining airway patency. A scalp incision and bur holes were created according to the entry points of the planned trajectory. An intraoperative microelectrode recording (MER) was routinely performed for each patient. A single-channel MER was performed using in an Alpha Omega recording system (Alpha Omega, Nazareth, Israel). The electrodes were advanced to 8 mm above the target using a clinical microdrive (microTargeting Drive; FHC Inc., Bowdoin, ME, USA). After MER, a quadripolar DBS electrode with a spacing of 0.5 mm between contacts (L301C, Beijing PINS Medical Co., China) was implanted into the target. The outer cannula was then pulled out and the electrode fixed. The same method was used for implantation on the contralateral side. Subsequently, an intraoperative MRI scan was performed and fused with preoperative planning to assess whether the electrodes were accurately implanted (Figure 3). Two extensions (E202C, Beijing PINS Medical Co., China) and a pulse generator (G102RS, Beijing PINS Medical Co., China) were then implanted.

### 2.5. Reconstruction of the DBS electrode positions

The pyramidal tract and DBS electrodes were reconstructed with Sinoplan software (SinovationMedical Technology Co. Ltd., Beijing China) using the post-operative CT and preoperative DTI. The pyramidal tract was from the primary motor cortex to the area of the corticospinal tract in the posterior limb of the internal capsule.

Actual locations of the DBS electrodes were reconstructed with Lead-DBS software (20). The post-operative CT data were linearly co-registered to the preoperative T1-weighted MRI images using advanced normalization tools and then normalized to the Montreal Neurological Institute space. The Hb was defined following the DBS Tractography Atlas (Middlebrooks 2020) provided by Lead-DBS (v2.5).

**Figure 4:**
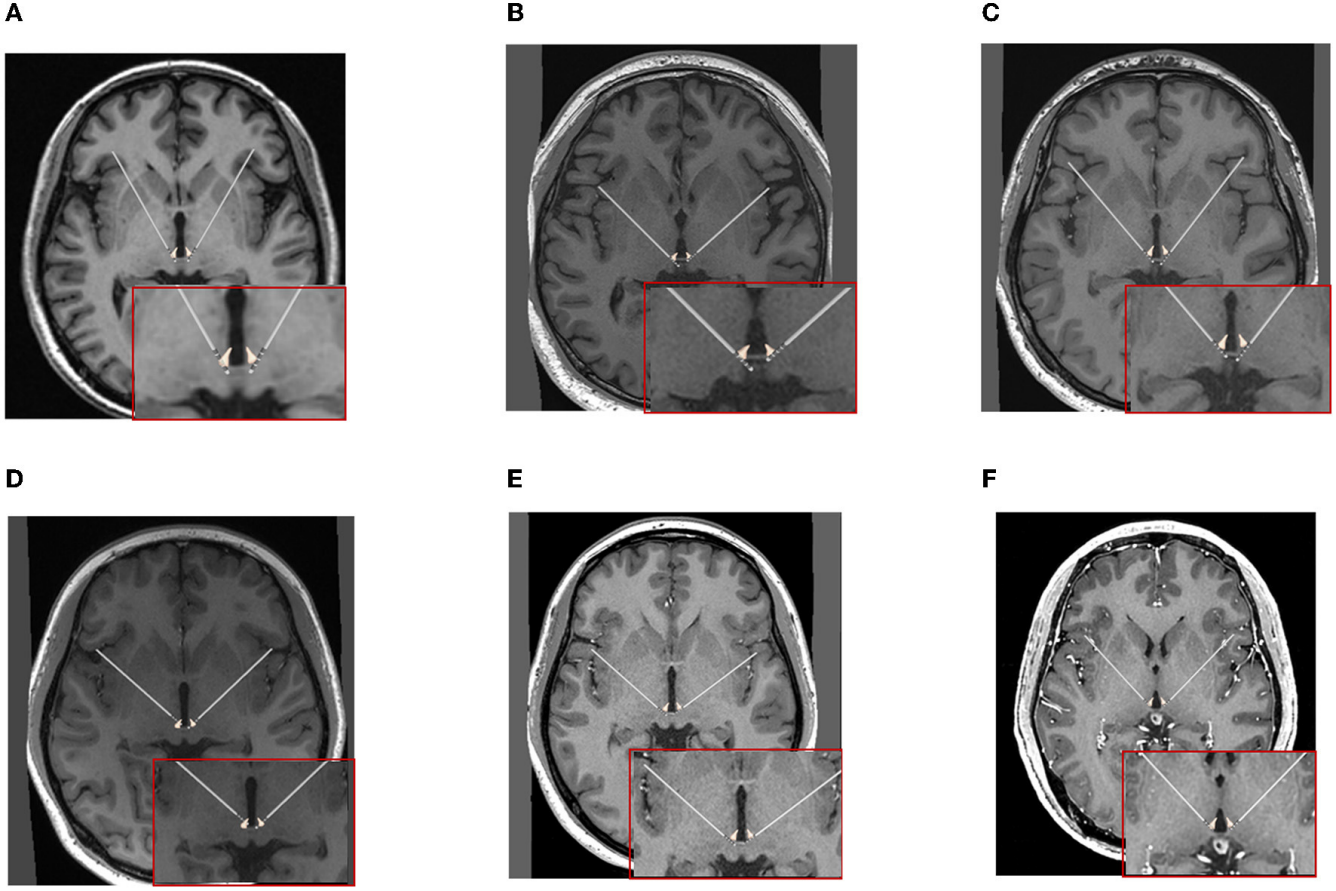
The positions of DBS electrodes were reconstructed with the preoperative MRI and CT at 1 month after surgery for each patient. **(A–F)** Cases 1–6.

The preoperative MRI and CT at 1 month after surgery were infused in the planning software. The actual coordinates of the most anterior end of the DBS electrode were used to calculate the differences from the planning (Figure 4).

### 2.6. Assessment of safety

**Figure 5:**
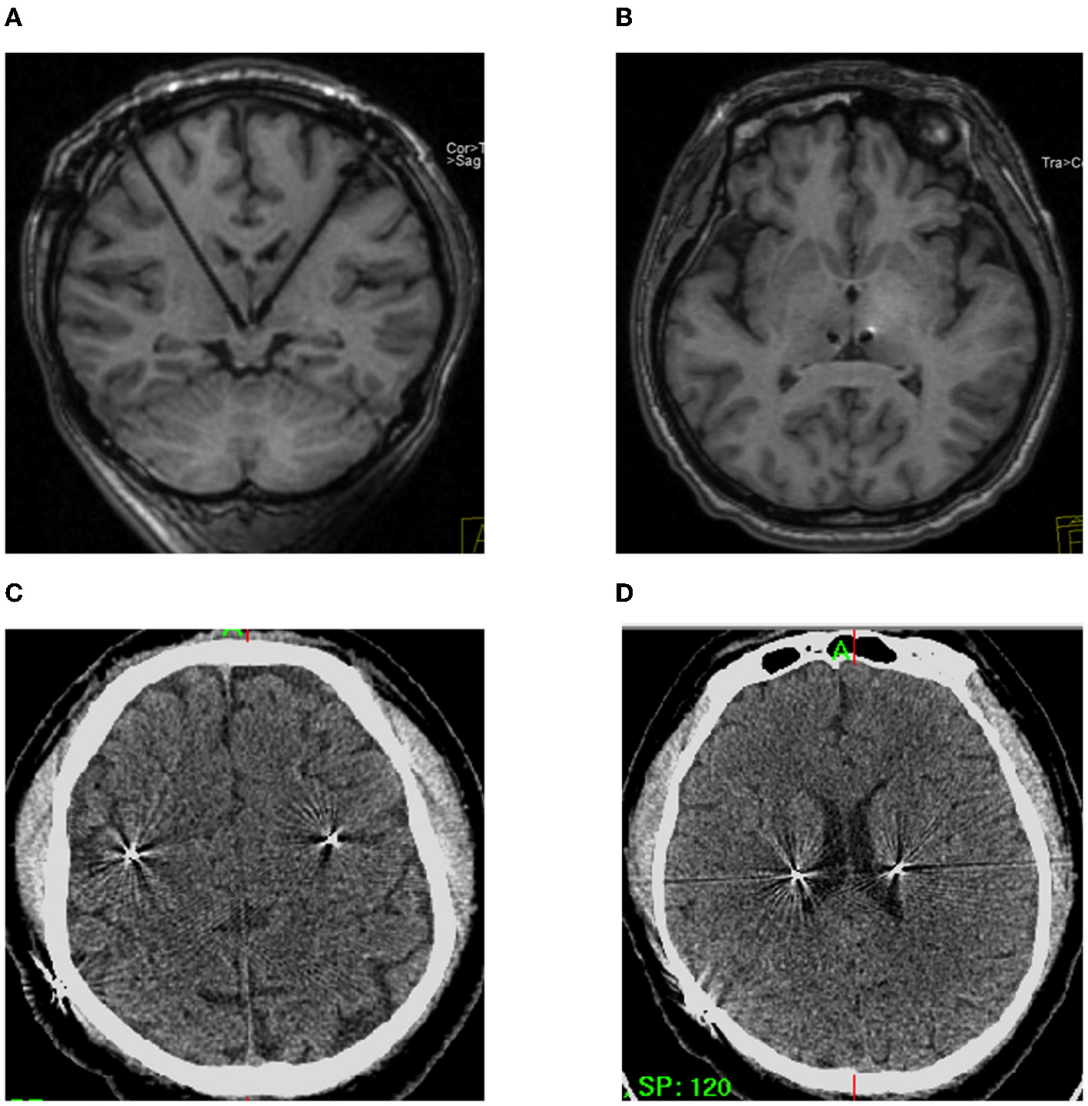
Safety of the frontal trajectory of the surgical path to the LHb. **(A, B)** No hematoma or ischemia as confirmed by intraoperative MRI. **(C, D)** Post-operative CT within 72 h on different planes.

There are three types of complications associated with LHb-DBS surgery, including complications related to the surgery, disease or device. Firstly, the surgery-related complications were assessed with the intraoperative MRI, post-operative CT within 72 h, and the reconstruction of pyramidal tract and DBS electrode (Figure 5; Supplementary Figure 2), including intracerebral hemorrhage, ischemia, infection, intracranial air, motor dysfunction and so on. Secondly, the disease -related complications were assessed by the psychiatrist, especially before turning on the stimulation, including depression, anxiety, suicide attempts, pain and so on. Thirdly, the device -related complications were assessed during intraoperative device testing, such as communication between the electrode or extension and the pulse generator.

### 2.7. Statistical analysis

Descriptive analyses were conducted with SPSS software (version 20, IBM Corporation, Armonk, New York, USA). Data were presented as the mean ± standard deviation (SD).

## 3. Results

### 3.1. Trajectory planning

The trajectory was individualized for the six patients. The Hb is visible in T1-weighted MR images (3.0T). The target coordinates (of the electrode tips) in the left LHb were 3.25 ± 0.82 mm (ranging 2.00 to 4.00 mm) laterally, 12.75 ± 0.42 mm (ranging 12.00–13.00 mm) posterior to the midpoint of the anterior commissure–posterior commissure (AC-PC) line, and 1.83 ± 0.68 mm (ranging 1.00–2.50 mm) inferior to the AC-PC line; whereas those in the right LHb were 3.25 ± 0.82 mm (ranging 2.00–4.00 mm) laterally, 13.00 ± 0.71 mm (ranging 12.00–14.00 mm) posterior to the midpoint of AC-PC line, and 1.17 ± 0.75 mm (ranging 0.00–2.00 mm) inferior to the AC-PC line. The “Ring” angles (relative to the AC-PC level on the sagittal section plane) of the trajectories to the left LHb and right LHb were 51.87° ± 6.67° (ranging 44.0°-59.1°) and 52.00° ± 7.18° (ranging 41.0°-58.9°), respectively (Figure 2C). The “Arc” angles (relative to the midline of sagittal plane) of the trajectories to the left LHb and right LHb were 33.82° ± 3.39° (ranging 27.7°-37.7°) and 33.55° ± 3.72° (ranging 27.0°-37.6°), respectively (Figure 2C). The target coordinates are given in Table 2.

**Table 2:** Target coordinates of the bilateral LHb in each patient.

**Case**	**Left**					**Right**				
	**X (mm)**	**Y (mm)**	**Z (mm)**	**Ring (**°**)**	**Arc (**°**)**	**X (mm)**	**Y (mm)**	**Z (mm)**	**Ring (**°**)**	**Arc (**°**)**
1	4.00	−13.00	1.00	44.00	27.70	4.00	−13.00	1.00	41.00	27.00
2	4.00	−13.00	2.50	49.10	35.30	4.00	−12.00	1.00	50.10	35.70
3	2.50	−13.00	1.00	45.00	35.00	2.50	−14.00	1.00	47.00	32.00
4	2.00	−12.00	2.00	59.10	37.70	2.00	−13.00	0.00	58.90	37.60
5	3.50	−13.00	2.50	57.20	34.40	3.50	−13.50	2.00	57.90	35.20
6	3.50	−12.50	2.00	56.80	32.80	3.50	−12.50	2.00	57.10	33.80

X, Y, Z, the coordinates of the most anterior end of DBS electrode of bilateral LHb in each patient.

### 3.2. Precision of frontal trajectory of the LHb

In the six patients, the intraoperative MRI with preoperative planning fused images showed that DBS electrodes were accurately implanted into the targets (as in the example shown in Figure 3). No patient underwent an intraoperative adjustment of an electrode. Moreover, using the fused images of the preoperative MRI and CT at 1 month after surgery, the reconstructed positions of the DBS electrodes are shown in Figure 4.

According to the mid-point of the AC-PC line, the actual coordinates of the DBS electrode tips in the left LHb were x′ ranging from +2.00 to +4.00 mm, y′ ranging from −9.10 to −14.10 mm, and z′ ranging from +0.30 to +2.40 mm. In the right LHb, x′ ranged from +1.50 to +3.80 mm, y′ ranged from −10.40 to −13.60 mm, and z′ ranged from −0.30 to 2.80 mm. The differences between the actual and planned coordinates of the electrode tips were calculated. In the case of the left LHb, Δx (the difference between x and x′) ranged from −0.50 to +1.70 mm, Δy (the difference between y and y′) ranged from −2.90 to +2.00 mm, and Δz (the difference between z and z′) ranged from −0.40 to +2.20 mm. In the case of the right LHb, Δx ranged from −0.80 to +2.00 mm, Δy ranged from −2.60 to +0.60 mm, and Δz ranged from −1.80 to +1.70 mm. The detailed results are given in Table 3.

**Table 3:** Actual implantation coordinates of the DBS electrode tips (mm) and differences from the target.

**Case**	**Left**						**Right**					
	**X**′	Δ**X**	**Y**′	Δ**Y**	**Z**′	Δ**Z**	**X**′	Δ**X**	**Y**′	Δ**Y**	**Z**′	Δ**Z**
1	4.00	0.30	−14.10	1.10	0.30	0.70	3.70	0.30	−13.60	0.60	0.50	0.50
2	4.00	1.70	−15.00	2.00	0.30	2.20	3.80	0.20	−12.20	−0.20	2.80	−1.80
3	2.50	0.30	−11.80	−1.20	0.80	0.20	2.60	−0.10	−13.10	−0.90	0.70	0.30
4	2.00	−0.50	−9.10	−2.90	2.40	−0.40	2.80	−0.80	−10.40	−2.60	1.50	−1.50
5	3.50	−0.10	−12.60	−0.40	1.00	1.50	1.50	2.00	−12.50	−1.00	−0.30	1.70
6	3.50	−0.40	−11.40	−1.10	1.10	0.90	3.20	0.30	−11.70	−0.80	0.60	1.40

### 3.3. Safety of LHb DBS surgery

Neither intraoperative MRI nor CT within 72 h after operation showed intracerebral hemorrhage or ischemia in the six patients (Figure 5). Additionally, the images showed that there was no intracranial pneumatosis. No patients had serious infections during the perioperative period. No device-related adverse events were observed. Moreover, no worsening depression, anxiety, suicide attempts, pain, or other disease-related complications were observed during the perioperative period, which defined as the time from admission to discharge. Four patients reported instantaneous electrical sensation when DBS was turned on. In addition, no surgery-related adverse events occurred during the 6 month follow-ups.

## 4. Discussion

### 4.1. Target coordinates of the LHb

The Hb comprises a pair of small nuclei adjacent to the posterior end of the medial dorsal thalamus and has a small volume of ~30 mm^3^ (21). The Hb can be identified in humans through high-resolution structural MRI (17). He et al. recently visualized the LHb adopting susceptibility-weighted imaging and quantitative susceptibility mapping (22). Detailed preoperative MR imaging evaluations are expected to facilitate more accurate implantations and interventions for the LHb. In our work, the Hb is visible in T1-weighted 3.0T MR images. The visualization of Hb improves the accuracy of target location. The design principle of the target is to maximize the area of contact between the electrode and the Hb while ensuring a safe implantation path. Therefore, in the present work, the target coordinates (electrode tips) were set in the posterior, medial, and inferior directions of the Hb. The target site was located ~12–14 mm posterior to the midpoint of the AC-PC line, 2–4 mm laterally, and 0–2.5 mm inferior to the (AC-PC) line. In Sartorius's paper (7), there was no detailed description of the target coordinates or surgical path. Schneider et al. measured functional coordinates in 27 DBS patients; the left lateral habenular complex (LHb-c) was located at *x* = −5.3 ± 1.69 mm, *y* = −11.8 ± 0.88 mm mid AC-PC, and *z* = 3.8 ± 0.68 mm and the right LHb-c was located at *x* = 5.6 ± 1.69 mm, *y* = −10.6 ± 0.9 mm mid AC-PC, and *z* = 3.9 ± 0.58 mm (18). However, these coordinates are the anatomical target coordinates of the LHb-c and not the electrode tip coordinates of the path, in contrast to our results. Additionally, Schneider et al. only planned the surgical planning system and did not test the system by performing surgery. Zhang et al. reported Hb-DBS for seven patients, with the target site located ~12–14 mm posterior to the midpoint of the AC-PC line, 2–4 mm laterally, and 0–2.5 mm inferior to the AC-PC line 15. They described coordinate ranges of the electrode tip that are basically consistent with ours but did not give the coordinates individually for each patient, and nor did they give the Arc and Ring angles of the path in detail. Therefore, we are the first to describe the target coordinates of the Hb-DBS electrode tip, providing a reliable reference for Hb-DBS surgery.

### 4.2. Trajectory planning design

Among the six patients, the “Ring” angle of the trajectories ranged from 41.0° to 59.1° relative to the axis plane and the Arc angle ranged from 27.0° to 37.7° relative to the sagittal plane. In three previous studies (7, 13, 15), there was no description of the Ring and Arc angles. Schneider et al. reported that a steep frontal trajectory (an angle <40° relative to the AC-PC line in sagittal images) for bilateral stimulation was possible in 96% of patients; this angle can avoid the superior thalamic vein, although a standard frontal trajectory (angle >40° relative to the AC-PC line in sagittal images) for bilateral stimulation was safely applicable in 48% of patients (18). Their results contrast with our results (ranging from 41.0° to 59.1°). There were three considerations in our trajectory planning. Firstly, according to measurements, the cortical entry point of the shortest path to the Hb located in the precentral gyrus. However, this entry point presents a danger in that if the puncture of the precentral gyrus and the surrounding cerebral cortex causes a hemorrhage or damage to the pyramidal tract, there might be sequelae of permanent hemiplegia. Considering safety, the cranial entry point was designed to be much as possible at the posterior middle frontal gyrus and as close as possible to the precentral gyrus. Secondly, if making a larger area of electrode contact with the LHb, the shortest path to the Hb should be with a smaller Arc angle relative to the midline of the sagittal plane. However, the trocar passing through the lateral ventricle leads to cerebrospinal fluid loss and brain displacement, reducing the precision of the implantation into the Hb. Therefore, the lateral ventricle was avoided and a larger arc was designed, with the Arc angle ranging from 27.0° to 37.7° relative to the sagittal plane in our work. Schneider et al. did not describe this angle. Thirdly, any trajectory planning targeting the Hb inevitably crosses the thalamus 18. In our patients, the trajectories passed through only the intralaminar nuclei, nuclei medialis, and lamina medullaris medialis.

Moreover, the following measures were taken to ensure the DBS electrodes reached the target accurately. Firstly, to ensure only slight outflow of cerebrospinal fluid, (1) the patient was generally anesthetized such that the position of the head was easy to adjust and the bone window was maximized to reduce the loss of cerebrospinal fluid, (2) a single-channel (instead of multi-channel) microelectrode recording was conducted for all patients to reduce the scope of dural damage, where bioglue was used to seal the dural rupture and bone holes and thus reduce the leakage of cerebrospinal fluid, and (3) the trocar did not puncture the lateral ventricle such that a large spillage of cerebrospinal fluid was avoided. Secondly, to ensure stable position of the lead in the brain parenchyma, the trocar was prevented from entering the lateral ventricle. Thirdly, intraoperative electrophysiology was applied to assist localization in each patient. We performed a single-channel microelectrode recording to help distinguish the discharge patterns between the thalamus and Hb (as in the example shown in Supplementary Figure 1). However, this study was the first to record the firing patterns of the Hb in humans; further analysis will be described elsewhere. Fourthly, the electrode positions were verified through real-time intraoperative MRI. After placing the stimulation electrodes, the intraoperative MR images were fused with the preoperative planning to check for deviation. In our surgeries, no patient underwent an intraoperative adjustment of an electrode. Moreover, the fused images of preoperative MRI and CT at 1 month after surgery showed that the DBS electrodes were implanted into the target with small errors.

### 4.3. Safety of LHb DBS surgery

Although DBS is generally a safe surgery for movement disorder patients, there are occasionally adverse events during surgery. The most serious adverse event is intracerebral hemorrhage, which has been reported with an estimated risk varying from 0.2 to 5.6% in functional neurosurgery for movement disorders (23–25). The risk of intracerebral hemorrhage strongly depends on how close the implanted DBS electrodes are to the blood vessels. In our frontal trajectory planning for each individual, the anterior caudate and putamen with abundant blood flow were well avoided when the DBS electrode was implanted at an angle >40° relative to the AC-PC line on the sagittal plane. Additionally, it has been reported that the superior thalamic vein is an important obstacle for the DBS trajectory of the Hb (18). Although the locations of thalamus-associated veins were not overviewed before surgery in our study, the images of intraoperative MRI and CT within 72 h after surgery showed there was no intracerebral hemorrhage in the six TRD patients, suggesting that our frontal trajectory poses little risk of intracerebral hemorrhage. Another serious side effect of DBS surgery is infection, including infection of the whole body and infection at the sites of the head incision and the pulse generator implantation. However, there were no infections in our six patients. This result may relate to the patients' young age and strong wound healing ability. Another notable side effect is the formation of intracranial air, which can squeeze the brain tissue, leading the brain to deform and shifting the coordinates of the original designed target. The main method adopted to avoid the formation of intracranial air is to reduce the loss of cerebrospinal fluid, as done well in our surgery. Intraoperative and post-operative MRI or CT images show that there was little intracranial air in all patients. Finally, the reconstruction results show that the implanted DBS electrode did not damage the pyramidal tracts (Supplementary Figure 2), which might cause severe motor dysfunction in patients. In all, frontal trajectories were successfully implemented for our six TRD patients, providing evidence that frontal trajectories are safe for LHb DBS.

Sartorius et al. reported initial weariness for several seconds in a patient with pacer activation, which might relate to interference with the intralaminar nuclei (7). Among our patients, only four reported transient inductance during DBS. However, the potential side effects of implantation paths will be of particular concern in follow ups.

A meta-analysis of 191 TRD patients treated with six DBS targets reported that common complaints included headache (26% patients), visual disturbances (21%), worsening depression (16%), sleep disturbances (16%), anxiety (14%), pain around incisions (9%), nausea (8%), device infection (8%), extension wires fractures (6%). The vast majority of these adverse events were transient and often resolved by adjusting stimulation parameter (26). In our study, implantations were accurately performed as planned for the six TRD patients without any serious complications, suggesting successful bilateral LHb DBS surgery. However, considering the small number of patients in the present study, the safety of LHb DBS should be further investigated.

### 4.4. Efficacy of LHb DBS in treating TRD

Owing to the long-standing monoaminergic hypothesis and pathologic neural circuits of depression, researchers are increasingly paying attention to LHb DBS for treating TRD (15, 16, 27–31). The five articles published to date mentioned only ~10 TRD patients treated with LHb DBS (7, 13–15, 19). The improvements in depression scores ranged from 11.1 to 100% (26). However, future trials and studies are required to confirm the efficacy and safety of LHb DBS in treating TRD. The efficacy and safety of LHb DBS for TRD will be followed continuously for our six patients.

### 4.5. Limitations

In our study, there still several limitations. Firstly, although there was not serious complications in the LHb DBS surgery, other fiber bundles associated with the frontal lobe were not fully considered, such as the dorsal component of the superior longitudinal fasciculus, the inferior fronto-occipital fasciculus, and the uncinated fasciculus and so on. Injury to these fiber bundles might affect patient's emotional or cognitive functions. Secondly, there was no detailed assessment of the patients' language or cognitive function before and after surgery, which was important to assess the safety of frontal trajectory in LHb DBS surgery.

## 5. Conclusions

Our results suggested that the frontal trajectory is safe, accurate, and feasible for LHb-DBS surgery. To the best of our knowledge, this is the first applicable work to report in detail the target coordinates and surgical path of human LHb-DBS. It has of great clinical reference value to treat more cases of LHb-DBS for TRD.

## Data availability statement

The original contributions presented in the study are included in the article/Supplementary material, further inquiries can be directed to the corresponding authors.

## Ethics statement

The studies involving human participants were reviewed and approved by Chinese People's Liberation Army General Hospital Medical Ethics Committee. The patients/participants provided their written informed consent to participate in this study. Written informed consent was obtained from the individual(s) for the publication of any potentially identifiable images or data included in this article.

## Author contributions

Conceptualization: ZW and LL. Data curation and funding acquisition: ZC and ZW. Formal analysis: ZC, ZW, and YT. Methodology: ZC, CJ, YT, ZL, JW, and TF. Project administration: CJ and CH. Resources: ZC, CJ, ZL, JW, and TF. Supervision: ZW and CH. Validation: ZC and CH. Roles and writing—original draft: ZC, CJ, and ZW. Writing—review and editing: ZW, HH, and LL. All authors contributed to the article and approved the submitted version.

## Supplementary material

Supplementary Figure 1 An example of a 4s single-channel microelectrode recording signals of the right Hb in patient 2. The discharge pattern at 2.135 mm is considered as belonging to the thalamus and the patterns at 1.850 and 0.604 mm belonging to the Hb. The discharge disappeared at −1.330 mm, considering the microelectrode was out of the Hb.

Supplementary Figure 2 Established positions of the pyramidal tract and DBS electrodes in case 3. **(A)** The front view shows the positions of left DBS electrodes and pyramidal tract. **(B)** The lateral view shows the positions of right DBS electrodes and the pyramidal tract.

## References

[1] Huang Y Wang Y Wang H Liu Z Yu X Yan J. Prevalence of mental disorders in China: a cross-sectional epidemiological study. Lancet Psychiatry. (2019) 6:211–24. 10.1016/S2215-0366(18)30511-X 30792114

[2] Lu J Xu X Huang Y Li T Ma C Xu G. Prevalence of depressive disorders and treatment in China: a cross-sectional epidemiological study. Lancet Psychiatry. (2021) 8:981–90. 10.1016/S2215-0366(21)00251-0 34559991

[3] Kisely S Li A Warren N Siskind D. A systematic review and meta-analysis of deep brain stimulation for depression. Depress Anxiety. (2018) 35:468–80. 10.1002/da.22746 29697875

[4] Mayberg HS Lozano AM Voon V McNeely HE Seminowicz D Hamani C. Deep brain stimulation for treatment-resistant depression. Neuron. (2005) 45:651–60. 10.1016/j.neuron.2005.02.014 15748841

[5] Malone DA Dougherty DD Rezai AR Carpenter LL Friehs GM Eskandar EN. Deep brain stimulation of the ventral capsule/ventral striatum for treatment-resistant depression. Biol Psychiatry. (2009) 65:267–75. 10.1016/j.biopsych.2008.08.029 18842257 PMC3486635

[6] Fenoy AJ Schulz PE Selvaraj S Burrows CL Zunta-Soares G. A longitudinal study on deep brain stimulation of the medial forebrain bundle for treatment-resistant depression. Transl Psychiatry. (2018) 8:111. 10.1038/s41398-018-0160-4 29867109 PMC5986795

[7] Sartorius A Kiening KL Kirsch P von Gall CC Haberkorn U Unterbergand AW. Remission of major depression under deep brain stimulation of the lateral habenula in a therapy-refractory patient. Biol Psychiatry. (2010) 67:e9–e11. 10.1016/j.biopsych.2009.08.027 19846068

[8] Holtzheimer PE Husain MM Lisanby SH Taylor SF Whitworth LA McClintock S. Subcallosal cingulate deep brain stimulation for treatment-resistant depression: a multisite, randomised, sham-controlled trial. Lancet Psychiatry. (2017) 4:839–49. 10.1016/S2215-0366(17)30371-1 28988904

[9] Coenen VA Bewernick BH Kayser S Kilian H Boström J Greschus S. Superolateral medial forebrain bundle deep brain stimulation in major depression: a gateway trial. Neuropsychopharmacology. (2019) 44:1224–32. 10.1038/s41386-019-0369-9 30867553 PMC6785007

[10] Bergfeld IO Mantione M Hoogendoorn ML Ruhé HG Notten P van Laarhoven J. Deep brain stimulation of the ventral anterior limb of the internal capsule for treatment-resistant depression: a randomized clinical trial. JAMA Psychiatry. (2016) 73:456–64. 10.1001/jamapsychiatry.2016.0152 27049915

[11] Clemm von Hohenberg C Weber-Fahr W Lebhardt P Ravi N Braun U Gass N. Lateral habenula perturbation reduces default-mode network connectivity in a rat model of depression. Transl Psychiatry. (2018) 8:68. 10.1038/s41398-018-0121-y 29581421 PMC5913319

[12] Yang Y Cui Y Sang K Dong Y Ni Z Ma S. Ketamine blocks bursting in the lateral habenula to rapidly relieve depression. Nature. (2018) 554:317–22. 10.1038/nature25509 29446381

[13] Wang ZY Cai XD Qiu RY Yao C Tian Y Gong C. Case report: lateral habenula deep brain stimulation for treatment-resistant depression. Front Psychiatry. (2021) 11:616501. 10.3389/fpsyt.2020.616501 33519557 PMC7838359

[14] Zhang C Kim SG Li D Zhang Y Li Y Husch A. Habenula deep brain stimulation for refractory bipolar disorder. Brain Stimul. (2019) 12:1298–300. 10.1016/j.brs.2019.05.010 31103455

[15] Zhang C Zhang Y Luo H Xu X Yuan TF Li D. Bilateral Habenula deep brain stimulation for treatment-resistant depression: clinical findings and electrophysiological features. Transl Psychiatry. (2022) 12:52. 10.1038/s41398-022-01818-z 35115488 PMC8813927

[16] Matsumoto M Hikosaka O. Lateral habenula as a source of negative reward signals in dopamine neurons. Nature. (2007) 447:1111–5. 10.1038/nature05860 17522629

[17] Lawson RP Drevets WC Roiser JP. Defining the habenula in human neuroimaging studies. Neuroimage. (2013) 64:722–7. 10.1016/j.neuroimage.2012.08.076 22986224 PMC3650642

[18] Schneider TM Beynon C Sartorius A Unterberg AW Kiening KL. Deep brain stimulation of the lateral habenular complex in treatment-resistant depression: traps and pitfalls of trajectory choice. Neurosurgery. (2013) 72(Suppl. 2):ons184-93; discussion ons193. 10.1227/NEU.0b013e318277a5aa 23147781

[19] Kiening K Sartorius A. A new translational target for deep brain stimulation to treat depression. EMBO Mol Med. (2013) 5:1151–3. 10.1002/emmm.201302947 23828711 PMC3944457

[20] Horn A Li NF Dembek TA Kappel A Boulay C. Lead-DBS v2: Towards a comprehensive pipeline for deep brain stimulation imaging. Neuroimage. (2019) 184:293–316. 10.1016/j.neuroimage.2018.08.068 30179717 PMC6286150

[21] Andres KH von Düring M Veh RW. Subnuclear organization of the rat habenular complexes. J Comp Neurol. (1999) 407:130–50. 10.1002/(SICI)1096-9861(19990428)407:1&lt;130::AID-CNE10&gt;3.0.CO;2-8 10213193

[22] He N Sethi SK Zhang C Li Y Chen Y. Visualizing the lateral habenula using susceptibility weighted imaging and quantitative susceptibility mapping. Magn Reson Imaging. (2020) 65:55–61. 10.1016/j.mri.2019.09.005 31655137

[23] Park JH Chung SJ Lee CS Jeon SR. Analysis of hemorrhagic risk factors during deep brain stimulation surgery for movement disorders: comparison of the circumferential paired and multiple electrode insertion methods. Acta Neurochir. (2011) 153:1573–8. 10.1007/s00701-011-0997-2 21476122

[24] Gorgulho A De Salles AA Frighetto L Behnke E. Incidence of hemorrhage associated with electrophysiological studies performed using macroelectrodes and microelectrodes in functional neurosurgery. J Neurosurg. (2005) 102:888–96. 10.3171/jns.2005.102.5.0888 15926715

[25] Cui ZQ Pan LS Liang SL Mao ZQ Xu X Yu XG. Early detection of cerebral ischemic events on intraoperative magnetic resonance imaging during surgical procedures for deep brain stimulation. Acta Neurochir. (2019) 161:1545–58. 10.1007/s00701-019-03929-x 31053908

[26] Hitti FL Yang AI Cristancho MA Baltuch GH. Deep brain stimulation is effective for treatment-resistant depression: a meta-analysis and meta-regression. J Clin Med. (2020) 9:2796. 10.3390/jcm9092796 32872572 PMC7564277

[27] Germann J Mameli M Elias GJB Loh A Taha A. Deep brain stimulation of the habenula: systematic review of the literature and clinical trial registries. Front Psychiatry. (2021) 12:730931. 10.3389/fpsyt.2021.730931 34484011 PMC8415908

[28] Sartorius A Henn FA. Deep brain stimulation of the lateral habenula in treatment resistant major depression. Med Hypotheses. (2007) 69:1305–8. 10.1016/j.mehy.2007.03.021 17498883

[29] Hong S Jhou TC Smith M Saleem KS Hikosaka O. Negative reward signals from the lateral habenula to dopamine neurons are mediated by rostromedial tegmental nucleus in primates. J Neurosci. (2011) 31:11457–71. 10.1523/JNEUROSCI.1384-11.2011 21832176 PMC3315151

[30] Tian J Uchida N. Habenula lesions reveal that multiple mechanisms underlie dopamine prediction errors. Neuron. (2015) 87:1304–16. 10.1016/j.neuron.2015.08.028 26365765 PMC4583356

[31] Elias GJB Germann J Loh A Boutet A Pancholi A. Habenular involvement in response to subcallosal cingulate deep brain stimulation for depression. Front Psychiatry. (2022) 13:810777. 10.3389/fpsyt.2022.810777 35185654 PMC8854862

