# Supplementary figures and images for: Safety and precision of frontal trajectory of lateral habenula deep brain stimulation surgery in treatment-resistant depression
Source: Front Neurol. 2023 Mar 16;14:1113545. doi: 10.3389/fneur.2023.1113545 (PMC10060811; doi:10.3389/fneur.2023.1113545)

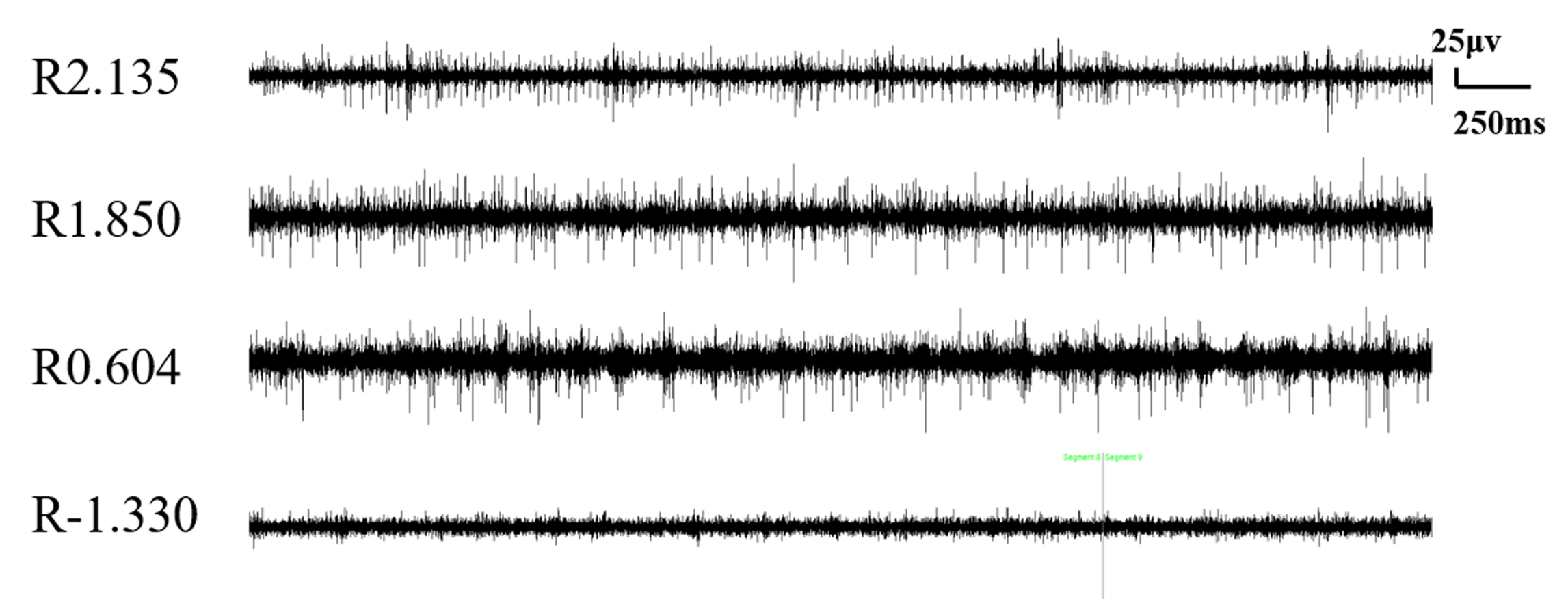

Supplement: Supplementary Figure 1 — An example of a 4s single-channel microelectrode recording signals of the right Hb in patient 2. The discharge pattern at 2.135 mm is considered as belonging to the thalamus and the patterns at 1.850 and 0.604 mm belonging to the Hb. The discharge disappeared at −1.330 mm, considering the microelectrode was out of the Hb. [file Image_1.TIF]

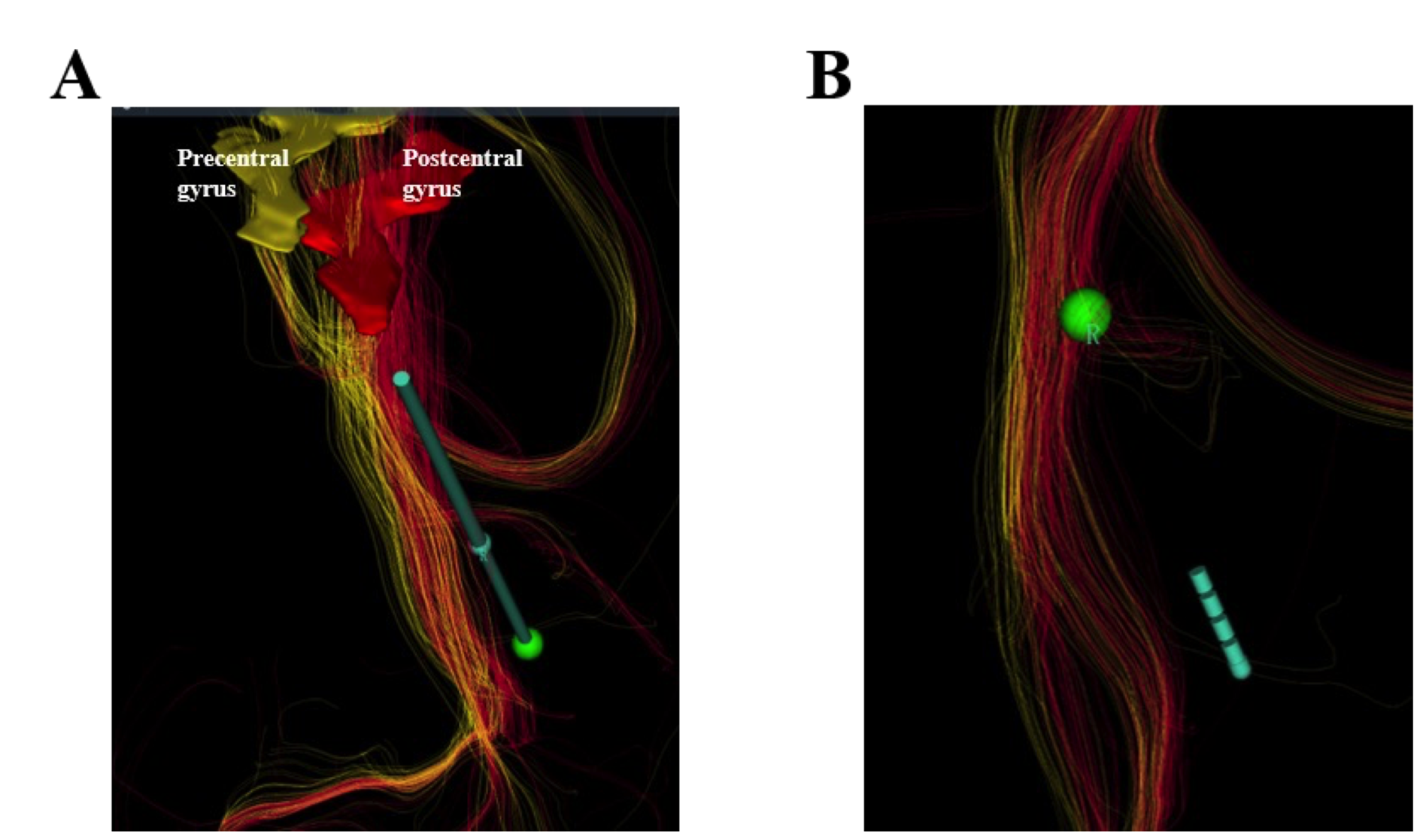

Supplement: Supplementary Figure 2 — Established positions of the pyramidal tract and DBS electrodes in case 3. (A) The front view shows the positions of left DBS electrodes and pyramidal tract. (B) The lateral view shows the positions of right DBS electrodes and the pyramidal tract. [file Image_2.TIFF]
